# Multidimensional employment precariousness mediates the association between low educational attainment and poor subjective well-being: results from a nationwide cross-sectional study in South Korea

**DOI:** 10.5271/sjweh.4109

**Published:** 2023-10-01

**Authors:** Seong-Uk Baek, Min-Seok Kim, Myeong-Hun Lim, Taeyeon Kim, Jong-Uk Won, Jin-Ha Yoon

**Affiliations:** 1 Department of Occupational and Environmental Medicine, Severance Hospital, Yonsei University College of Medicine, Seoul, Republic of Korea.; 2 The Institute for Occupational Health, Yonsei University College of Medicine, Seoul, Republic of Korea.; 3 Graduate School, Yonsei University College of Medicine, Seoul, Republic of Korea.; 4 Department of Public Health, Graduate School, Yonsei University College of Medicine, Seoul, Republic of Korea.; 5 Department of Preventive Medicine, Yonsei University College of Medicine, Seoul, Republic of Korea.

**Keywords:** decent work, employment quality, inequality, mental health, precarious employment, precarious work

## Abstract

**Objective:**

This paper explored how multidimensional employment precariousness (MEP) mediates the relationship between educational attainment and subjective well-being.

**Methods:**

A nationwide sample of 46 919 Korean workers participated in surveys between 2017 and 2020. Educational attainment was divided into four categories: elementary school, middle school, high school, and college. Subjective well-being was assessed using the 5-item World Health Organization Well-Being Index, and MEP was evaluated using a modified version of the Employment Precariousness Scale (ERPES-E), with scores ranging from 0 to 100 and higher scores indicating worse employment precariousness. A counterfactual-based logistic mediation analyses were used to estimation.

**Results:**

The mean MEP score was 36.0 [standard deviation (SD) 12.1] for college education, 44.3 (SD 11.5) for high school, 49.5 (SD 10.1) for middle school, and 51.1 (SD 10.0) for elementary school. The prevalence of poor subjective well-being was 24.0% for college education, 31.3% for high school, 40.6% for middle school, and 44.8% for elementary school. Odds ratios (OR) for the total effect of education on the poor subjective well-being were 1.44 [95% confidence interval (CI) 1.37–1.53] for high school, 2.19 (95% CI 1.98–2.24) for middle school, and 2.40 (95% CI 2.04–2.82) for elementary school when compared to college education. The OR for the indirect effect mediated through MEP were 1.27 (95% CI 1.25–1.29) for high school, 1.46 (95% CI 1.42–1.51) for middle school, and 1.53 (95% CI 1.48–1.59) for elementary school, accounting for 63.9%, 48.5%, and 48.6% of the total effect, respectively.

**Conclusion:**

Our study suggests that MEP is an important contributor to the disparities in subjective well-being resulting from educational gradients.

Globally, there has been an increasing social interest in precarious employment in recent times. The standard employment relationship generally refers to an employment condition in which workers are part of a stable, full-time, and permanent labor contract while enjoying extensive legal rights and benefits (1). However, the advent of the Fourth Industrial Revolution and the digitalization of labor have brought about changes in the labor market (2). The recent COVID-19 pandemic has also accelerated the weakening of the standard employment relationship. The labor market witnessed a notable upsurge in the adoption of flexible work arrangements, such as freelancers and gig workers (3). Moreover, the pandemic has caused more workers to move into more precarious and low-paying job positions, disproportionately affecting women and unskilled workers (4). While precarious employment primarily referred to temporary employment in the past, the rapid transformation of the labor market now demands that researchers conceptualize and measure precarious employment using a multidimensional approach (5, 6). Compared to a unidimensional approach that classifies precarious employment solely based on job insecurity or type of contract, a multidimensional approach has gained increasing importance in epidemiological research because it has been found to be more sensitive to workers’ health outcomes (7). Since the initial attempt by Amable et al (8) to conceptualize precarious employment as a multidimensional concept, measurement tools such as the Employment Precariousness Scale (ERPES) have been developed and widely used in various epidemiological studies (9). While there is no consensus on the definition of multidimensional employment precariousness (MEP), studies have proposed that it consists of various elements, including temporary employment, income inadequacy, and a lack of rights and protection (10, 11). This multidimensional approach represents a worker’s level of precariousness as a specific point on a continuous spectrum rather than relying on the traditional binary categorization of temporary versus permanent employment. The typological approach, as an alternative methodology, has employed latent class analysis to classify the multidimensional characteristics of precarious employment among workers, revealing diverse typologies of MEP across regions and countries (6, 12, 13).

Recent studies have shown that workers with precarious employment are associated with various negative health outcomes, including a higher body mass index (14), a higher incidence of cardiovascular disease and stroke (15), and a higher overall mortality (16). Aside from physical health, high levels of MEP have consistently been found to be associated with poor mental health, including psychological distress, depressive symptoms, and suicidal ideation (17–20). Although all individual aspects of MEP may affect the psychological health of workers, employment insecurity, low wages, and vulnerability have specifically been demonstrated to have a strong correlation with adverse mental health outcomes for workers (21, 22).

It is important to recognize that decent work is not equally available to all workers and that certain individuals are more likely to experience precarious employment than others. Vulnerable groups, including women, ethnic minorities, and those with low educational attainment, are particularly susceptible to high levels of precariousness (23). Employers often seek employees with higher levels of education, as this can indicate greater qualifications and the potential for better work performance (24). Indeed, previous studies have consistently shown that individuals with low educational attainment are exposed to higher levels of MEP (6, 23, 25). Consequently, unequal access to higher education during adolescence and young adulthood has been believed to cause social inequalities later in life (26), given that individuals with lower levels of education are disproportionately allocated to jobs with insecure and hazardous conditions and are rewarded poorly.

Researchers in the field of public health consider educational attainment to be one of the critical social determinants of mental health (27). In the Korean context, there has been significant improvement in overall educational attainment over the past few decades. Despite this advancement, social inequality driven by educational disparities persists in Korean society and is recognized as an important determinant of mental health (28). From the perspective of precarious employment, previous studies have demonstrated that workers with low educational attainment are over-represented in part-time and temporary employment in Korea, experiencing high employment insecurity (29, 30). These types of jobs were found be associated with poor psychological health, including depression and low subjective well-being (29, 30). However, most existing Korean literature investigating the association between education and precarious employment, or between precarious employment and health, has primarily defined precarious employment solely based on contract types, while multidimensional approaches incorporating factors such as workers’ rights and vulnerability are scarce (31).

Previous studies have shown that low educational attainment is associated with mental health problems (32, 33). However, individuals with low educational attainment are often exposed to multiple risk factors for poor mental health, making it difficult to identify the underlying causes. Several studies have explored the mediating role of occupational factors in the relationship between educational attainment and mental health. For instance, one Australian study has suggested that occupational factors, such as psychosocial job quality, insecure employment relationship, and income can mediate the association between educational attainment and mental health (34). Similarly, previous US studies have demonstrated that the psychosocial work environment or employment quality can act as a mediator in the education–mental health relationship (35, 36). However, there have been no studies examining how occupational experiences and exposures mediate the relationship between education level and mental health in the context of Korea or the broader East Asian region. Furthermore, there is a lack of prior research specifically discussing this mediating effect based on the framework of precarious employment.

In this study, we aimed to examine the mediating effect of MEP on the relationship between low educational attainment and poor subjective well-being.

## Methods

### Study population

The study sample was drawn from the 5^th^ and 6^th^ Korean Working Conditions Surveys (KWCS), which were conducted in 2017 and 2020, respectively. The KWCS is a nationwide repeated cross-sectional study, which the Occupational Safety and Health Research Institute (OSHRI) conducts every three years. The KWCS was designed to include a nationally representative sample of approximately 50 000 South Korean workers. It uses a systematic sampling method to select the study sample, in which an enumeration district in South Korea serves as the primary sampling unit and households and household members as the secondary sampling units. The KWCS constructs the content of its questionnaire by referring to the content of the European Working Conditions Survey (EWCS), with input from experts in the field of occupational safety and health in Korea (37). As the related questions about MEP were collected beginning with the 5^th^ KWCS, our analysis included the study population of the 5^th^ and 6^th^ KWCS. The 5^th^ KWCS was conducted from July to November 2017 and the 6^th^ KWCS was conducted from October 2020 to April 2021.

**Figure 1:**
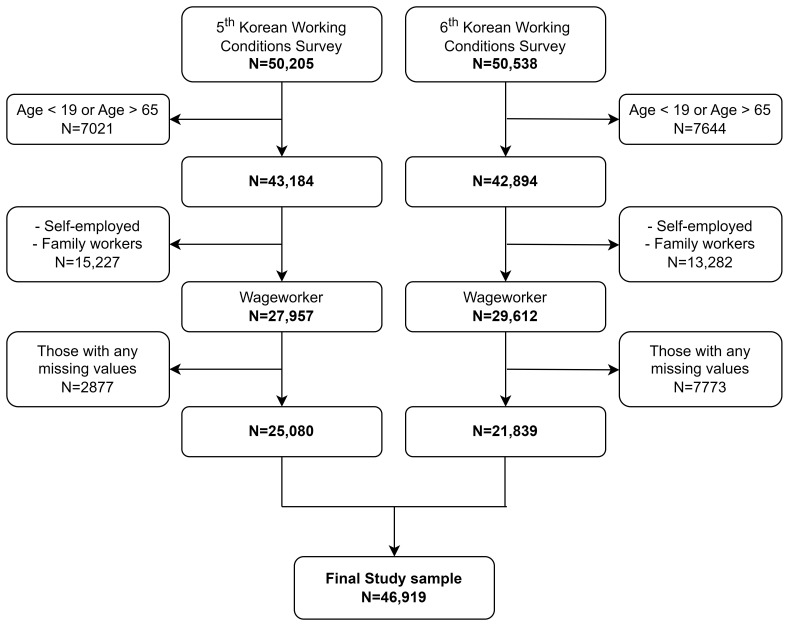
Flowchart of the selection of study participants.

Figure 1 depicts a flowchart of the study sample selection process. The inclusion criteria were as follows: (i) age 19–65 years, (ii) wage workers (salaried workers), and (iii) no missing values for any variables. For the purpose of pooled cross-sectional analysis, a final sample of 46 919 workers (25 080 workers from the 5^th^ KWCS and 21 839 workers from the 6^th^ KWCS) was assembled for the main analysis.

### Data availability and ethics statement

Raw KWCS data can be obtained at https://oshri.kosha.or.kr/oshri. The Institutional Review Board of authors’ institution approved this study (4–2021–1303).

### Variables

*Independent variable (educational attainment).* All survey participants were asked “What is the highest level of education that you have completed?” Possible answers were: “No education or lower than elementary school,” “Elementary school (primary education),” “Middle school (lower secondary education),” “High school (upper secondary education),” “Community college,” “University-undergraduate,” “Graduate or above” The regular education system for Koreans uses a 6-3-3-4 single ladder system, which consists of 6-year elementary education, 3-year middle school education, 3-year high school education, and 4-year college or university. In line with this framework, respondents’ educational attainment was categorized into four groups: “Elementary school or below,” “Middle school,” “High school,” and “College or above.” This classification aligns with previous Korean studies and is culturally appropriate within the Korean context (38, 39). We considered respondents whose educational attainment was college or above to be the reference group.

*Mediating variable (MEP).* The MEP measurement used in this study was originally developed by Padrosa et al (5) as an adaptation of the Employment Precariousness Scale for Europe, namely the EPRES-E. Previous studies have confirmed the reliability and validity of the measurement (5, 40). While the EPRES is a construct that originally consisted of six dimensions, namely “temporariness” “disempowerment,” “vulnerability,” “wages,” “rights,” and “exercise of rights,” the EPRES-E dropped the dimension of “rights” and added a new one, that is “uncertain working hours.” Each dimension is measured by two or three items (proxy indicators), each of which is measured using 3–5-point ordinal scales: (i) temporariness: “duration of contract,” “tenure”; (ii) disempowerment: “trade unions,” “meetings”; (iii) vulnerability: “respect of boss,” “fair treatment”; (iv) exercise of rights: “utilizing break,” “hours off for personal matters”; (v) uncertain working times: “schedule unpredictability,” “work at short notice,” “working times regularity”; and (vi) wages: “net earning per month,” “net earning per hour”. Regarding scoring of EPRES-E, for instance, the dimension “temporariness” consists of two proxy indicators: “duration of contract” (4-point scale) and “tenure” (3-point scale). The EPRES-E gives the same weight to each component of the instrument and thereby the scoring for each dimension was devised to be averages of the individual items, which were transformed into a 0–100 scale, with a higher score indicating a higher level of precarity. The total score is again calculated as the average score across all six dimensions (5). The KWCS is designed to have the same item composition as the EWCS and occupational safety and health experts participate in the translation of the questionnaire of the EWCS (37). This enabled the application of the same operationalization of EPRES-E in the content of the KWCS. The detailed questionnaire can be obtained from the study conducted by Padrosa et al (5). Previous research has explored the adaptability of EPRES-E in measuring precarious employment within the context of the Korean labor market and has demonstrated the close relationship between each dimension of EPRES-E and subjective well-being of Korean workers (31).

*Dependent variable (subjective well-being).* We employed the 5-item World Health Organization Well-Being Index (WHO-5 index) to measure the workers’ subjective well-being. The WHO-5 index consists of five items assessing the participants’ overall psychological well-being over the last two weeks. The specific items of the WHO-5 index were: (i) “I have felt cheerful and in good spirits”; (ii) “I have felt calm and relaxed”; (iii) “I have felt active and vigorous”; (iv) “I woke up feeling fresh and rested”; and (v) “My daily life has been filled with things that interest me”. The scoring for each item ranged from 0 (none of the time) to 5 (all of the time). The WHO-5 index score was defined as the total sum of scores multiplied by 4, and ranged from 0–100. Previous studies have confirmed the reliability and validity of the WHO-5 index, which is widely used for depression screening (41). Following the results of an earlier study, we defined individuals whose WHO-5 index score was <50 as having poor subjective well-being (41).

### Covariates

We considered the following covariates as potential confounders. Gender (men/women) was adjusted. Age was categorized as 19–29, 30–39, 40–49, 50–59, and 60–65 years. Residential area was categorized as metropolitan and small cities/rural. Occupation was categorized as (i) blue collar, (ii) service and sales workers, and (iii) white collar according to the Korean Standard Classification of Occupations. Marital status was categorized as married versus unmarried or other (divorced, separated, widowed). Participants were asked whether they had a health problem or disease that had lasted or was likely to last >6 months. Respondents who answered “yes” were classified as having a chronic disease, while those who answered “no” were classified as not having a chronic disease. Survey year (2017 or 2020) was adjusted.

### Statistical analysis

*Descriptive analysis.* Statistical analysis was performed separately for each group, including the overall, male, and female samples. We examined the differences in the distribution of characteristics and MEP according to the respondents’ educational attainment. Next, the distribution of MEP and prevalence of poor subjective well-being according to study variables were calculated.

*Preliminary analysis.* As a preliminary analysis, we examined whether there were any associations between the two indirect paths using multivariate linear or logistic regression. Specifically, the following associations were explored: (i) the association between educational attainment and MEP (educational attainment → MEP score) and (ii) the association between MEP score and poor subjective well-being (MEP score → poor subjective well-being). Additionally, to examine whether the association varies by gender, a model with interaction terms for gender was fitted for each pathway. Specifically, interaction terms between exposure (educational attainment) and gender and between mediator (MEP) and gender were included.

**Figure 2:**
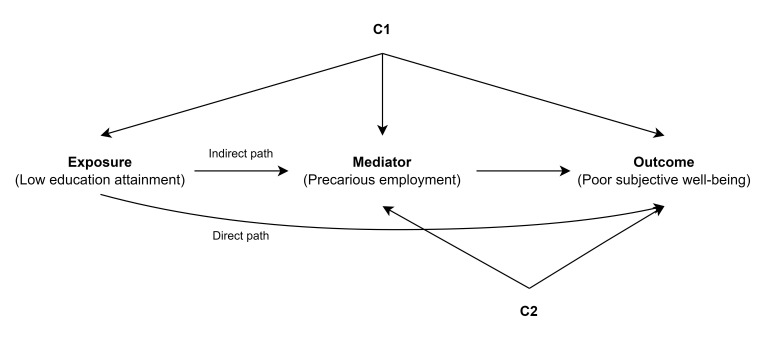
Directed acyclic graph for the assumed causal relationship between educational attainment (exposure), and poor subjective well-being (outcome), mediated through precarious employment (mediator). The observed confounder C1 includes gender, age, and residential area, and observed confounder C2 includes marital status, occupation, chronic disease, and survey year.

*Mediation analysis.* Our mediation analysis was based on the following causal assumption of the two main paths linking the educational attainment and poor subjective well-being, as depicted in figure 2. The first is the direct path, in which low educational attainment is associated with poor subjective well-being, regardless of employment precariousness. The second is the indirect path, in which low educational attainment is associated with poor subjective well-being, because low educational attainment is associated with a high level of employment precariousness. We conducted a simple counterfactual-based mediation analysis using a method proposed by Buis (42). The decomposition of the total effect into the direct effect and indirect effect was conducted within the potential outcomes framework, as detailed in the supplementary materials (see supplementary details). Our primary estimands of interest, namely the natural indirect effect, compare the odds of poor subjective well-being under the MEP level that would arise with and without the exposure condition (low educational attainment) within the same exposure group. The direct effect compares the odds of poor subjective well-being corresponding to a specific educational status versus the reference status, while keeping the distribution of MEP levels constant. The effect size was presented as odds ratios (OR) with 95% confidence intervals (CI). We did not hypothesize the exposure–mediator interaction. We employed 1000 bootstrap resampling to estimate the CI. The proportion mediated was calculated by dividing indirect effects by total effect.

We pooled cross-sectional samples from 2017 and 2020 to explore the overall association between educational attainment, precarious employment, and subjective well-being. Multivariate linear or logistic regression in the preliminary analysis was performed using R software (version 4.2.3; R Foundation for Statistical Computing, Vienna, Austria). Causal mediation analysis was performed using “*ldecomp*” package (42) in Stata (version 18.0; StataCorp LLC, College Station, TX, USA). Visualization was performed using R.

*Sensitivity analysis.* First, we calculated the mediational E-value that represents the magnitude by which an unobserved confounding would need to influence both the mediator and the outcome in order to completely nullify the mediational effect (indirect effect) (43, 44). Second, we used multiple imputation to address missing values and the analyses were repeated. Third, the analysis was repeated separately for 2017 and 2020, considering that COVID-19 has profoundly affect the characteristics of the precarious employment in labor market (45). For multiple imputation, 20 imputed datasets without missing values were generated through a chained-equation method under missing-at-random (MAR) assumption.

## Results

### Descriptive analysis

Among the total sample of 46 919 participants, 27 129 (57.8%) had completed a college education or above, 16 812 (35.8%) had completed high school education, 2243 (4.8%) had completed middle school education, and 735 (1.6%) had completed elementary school education (table 1). A higher proportion of workers in the older age groups (50–59 and 60–65 years), residing in small cities/rural areas, unmarried, engaged in blue-collar jobs, and having chronic diseases was observed in the group with lower educational levels compared to the group with a college education.

**Table 1:** Characteristics of the study population stratified by educational attainment.

Characteristics		Men								Women						
		College (N=13 889)		High school (N=7429)		Middle school (N=878)		Elementary school (N=257)		College (N= 13 240)		High school (N= 9383)		Middle school (N= 1365)		Elementary school (N=478)
		N (%)		N (%)		N (%)		N (%)		N (%)		N (%)		N (%)		N (%)
Age group (years)																
	19–29	2016 (14.5)		1383 (18.6)		22 (2.5)		5 (1.9)		2569 (19.4)		987 (10.5)		18 (1.3)		5 (1.0)
	30–39	4903 (35.3)		1114 (15.0)		36 (4.1)		11 (4.3)		4391 (33.2)		926 (9.9)		15 (1.1)		11 (2.3)
	40–49	4168 (30.0)		1756 (23.6)		84 (9.6)		13 (5.1)		4090 (30.9)		2481 (26.4)		64 (4.7)		11 (2.3)
	50–59	2356 (17.0)		2222 (29.9)		330 (37.6)		92 (35.8)		2017 (15.2)		4036 (43.0)		577 (42.3)		137 (28.7)
	60–65	446 (3.2)		954 (12.8)		406 (46.2)		136 (52.9)		173 (1.3)		953 (10.2)		691 (50.6)		314 (65.7)
Residential area																
	Metropolitan	7064 (50.9)		3588 (48.3)		416 (47.4)		86 (33.5)		6982 (52.7)		4688 (50.0)		629 (46.1)		184 (38.5)
	Small cities/rural	6825 (49.1)		3841 (51.7)		462 (52.6)		171 (66.5)		6258 (47.3)		4695 (50.0)		736 (53.9)		294 (61.5)
Marital status																
	Married	9568 (68.9)		4349 (58.5)		507 (57.7)		145 (56.4)		9029 (68.2)		6364 (67.8)		800 (58.6)		251 (52.5)
	Unmarried or others	4321 (31.1)		3080 (41.5)		371 (42.3)		112 (43.6)		4211 (31.8)		3019 (32.2)		565 (41.4)		227 (47.5)
Occupation																
	Blue collar	3111 (22.4)		5197 (70.0)		816 (92.9)		235 (91.4)		544 (4.1)		2507 (26.7)		820 (60.1)		335 (70.1)
	Service and sales worker	2131 (15.3)		1349 (18.2)		35 (4.0)		11 (4.3)		2913 (22.0)		5213 (55.6)		528 (38.7)		127 (26.6)
	White collar	8647 (62.3)		883 (11.9)		27 (3.1)		11 (4.3)		9783 (73.9)		1663 (17.7)		17 (1.2)		16 (3.3)
Chronic disease																
	Yes	539 (3.9)		417 (5.6)		85 (9.7)		49 (19.1)		443 (3.3)		514 (5.5)		161 (11.8)		122 (25.5)
	No	13 350 (96.1)		7012 (94.4)		793 (90.3)		208 (80.9)		12 797 (96.7)		8869 (94.5)		1204 (88.2)		356 (74.5)
Survey year																
	2017	7433 (53.5)		3893 (52.4)		544 (62.0)		156 (60.7)		6865 (51.9)		5086 (54.2)		818 (59.9)		285 (59.6)
	2020	6456 (46.5)		3536 (47.6)		334 (38.0)		101 (39.3)		6375 (48.1)		4297 (45.8)		547 (40.1)		193 (40.4)

**Figure 3:**
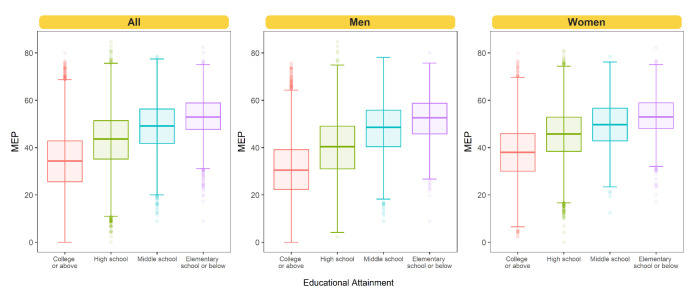
Distribution of multidimensional employment precariousness (MEP) according to educational attainment. Values that are either 1.5 times the interquartile range (IQR) above the upper quartile or 1.5 times the IQR below the lower quartile were presented as outliers.

The mean MEP was 39.9 [standard deviation (SD) 12.7] for all workers, 36.4 (SD 13.0) for men, and 43.1 (SD 11.4) for women (see supplementary material, https://www.sjweh.fi/article/4109, figure S1). The mean MEP was 36.0 (SD 12.1) for college or above, 44.3 (SD 11.5) for high school, 49.5 (SD 10.1) for middle school, and 51.1 (SD 10.0) for elementary school or below in overall sample (figure 3). The mean MEP was higher among young aged workers aged <30 (46.2) years and older aged workers aged ≥60 (45.0) years compared to middle-aged workers. Additionally, the mean MEP was higher among workers with blue-collar jobs (42.9) or service/sales workers (45.7), compared to workers with white-collar jobs (34.6) (supplementary table S1). The prevalence of poor subjective well-being was 24.0% for college or above, 31.3% for high school, 40.6% for middle school, and 44.8% for elementary school or below (supplementary table S2). The prevalence of high educational attainment (college or above) was higher among those without poor subjective well-being (60.8%), compared to those with poor well-being (50.0%) (supplementary table S3).

### Preliminary analysis

For the first indirect path (education attainment → MEP), lower educational attainment was associated with an increase in MEP score [high school β=4.93 (95% CI 4.69–5.17); middle school β =9.10 (95% CI 8.59–9.61); elementary school or below β=10.14 (95% CI 9.34–10.94)] in the overall sample (table 2). For male workers, the association between educational attainment and MEP was β=5.46 (95% CI 5.10–5.81) for high school, β=11.63 (95% CI 10.83–12.42) for middle school, and β=13.26 (95% CI 11.92–14.60) for elementary school or below compared to college education. For female workers, the association between educational attainment and MEP score was β=3.86 (95% CI 3.54–4.19) for high school, β=6.89 (95% CI 6.24–7.55) for middle school, and β=8.12 (95% CI 7.14–9.10) for elementary school or below. For the second indirect path (MEP → poor subjective well-being), the OR of the association between a 1-point increase in MEP and poor subjective well-being was 1.03 (95% CI 1.03–1.03) in the overall, male, and female samples. In adjusted models, interaction terms between gender and educational attainment indicated that gender modifies the educational attainment-MEP relationship (supplementary table S4), with smaller differences in MEP across the educational gradient among women compared to men.

**Table 2:** Association of education attainment with multidimensional employment precariousness (MEP) and MEP with poor subjective well-being. [OR=odds ratio; CI=confidence interval].

Path			All				Men				Women		
			β	OR	95% CI		β	OR	95% CI		β	OR	95% CI
Model 1 ^a^													
	Educational attainment → MEP												
		College or above	0.00		Reference		0.00		Reference		0.00		Reference
		High school	4.93		4.69–5.17		5.46		5.10–5.81		3.86		3.54–4.19
		Middle school	9.10		8.59–9.61		11.63		10.83–12.42		6.89		6.24–7.55
		Elementary school or below	10.14		9.34–10.94		13.26		11.92–14.60		8.12		7.14–9.10
Model 2 ^a^													
	MEP → poor subjective well-being												
		MEP (range 0–100)		1.03	1.03–1.03			1.03	1.03–1.03			1.03	1.03–1.03

^a^ Adjusted for gender, age, residential area, marital status, occupation, chronic disease, and survey year.

### Mediation analysis

The total, direct, and indirect effects of educational attainment on poor subjective well-being increased with lower levels of education, indicating a dose–response relationship in overall sample. For overall workers, the OR of the indirect effect was 1.27 (95% CI 1.25–1.29) for high school, 1.46 (95% CI 1.42–1.51) for middle school, and 1.53 (95% CI 1.48–1.59) for elementary school or below, accounting for 63.9%, 48.5%, and 48.6% of the total effect, respectively (table 3). For male workers, the OR of the indirect effect was 1.31 (95% CI 1.28–1.35) for high school, 1.59 (95% CI 1.52–1.67) for middle school, and 1.69 (95% CI 1.59–1.79) for elementary school or below, accounting for 57.8%, 52.0%, and 58.5% of the total effect, respectively. For female workers, the OR the indirect effect was 1.22 (95% CI 1.20–1.25) for high school, 1.36 (95% CI 1.32–1.41) for middle school, and 1.41 (95% CI 1.35–1.47) for elementary school or below, accounting for 72.7%, 45.7%, and 42.4% of the total effect, respectively. The findings showed that as the level of education decreases, the ORs of the indirect effect increased.

**Table 3:** Mediating effect of multidimensional employment precariousness on the association between low educational attainment and poor subjective well-being. Models adjusted for gender, age, residential area, marital status, occupation, chronic disease, and survey year. [OR=odds ratio; CI=confidence interval]

Educational attainment		Total effect		Direct effect		Indirect effect		Proportion mediated
		OR (95% CI)		OR (95% CI)		OR (95% CI)		% (95% CI)
All								
	College or above	Reference		Reference		Reference		Reference
	High school	1.44 (1.37–1.53)		1.14 (1.08–1.20)		1.27 (1.25–1.29)		63.9 (54.2–73.6)
	Middle school	2.19 (1.98–2.43)		1.50 (1.35–1.66)		1.46 (1.42–1.51)		48.5 (41.8–55.3)
	Elementary school or below	2.40 (2.04–2.82)		1.57 (1.33–1.84)		1.53 (1.48–1.59)		48.6 (39.1–58.2)
Men								
	College or above	Reference		Reference		Reference		Reference
	High school	1.60 (1.49–1.72)		1.22 (1.13–1.31)		1.31 (1.28–1.35)		57.8 (47.8–67.8)
	Middle school	2.46 (2.10–2.87)		1.54 (1.32–1.80)		1.59 (1.52–1.67)		52.0 (42.0–61.9)
	Elementary school or below	2.44 (1.87–3.19)		1.45 (1.12–1.88)		1.69 (1.59–1.79)		58.5 (38.9–78.1)
Women								
	College or above	Reference		Reference		Reference		Reference
	High school	1.32 (1.22–1.42)		1.07 (1.00–1.16)		1.22 (1.20–1.25)		72.7 (51.0–94.4)
	Middle school	1.96 (1.71–2.25)		1.44 (1.26–1.65)		1.36 (1.32–1.41)		45.7 (35.3–55.9)
	Elementary school or below	2.25 (1.83–2.77)		1.60 (1.30–1.96)		1.41 (1.35–1.47)		42.4 (30.3–54.5)

### Sensitivity analysis

The E-values of the indirect effects 1.51 (lower bound: 1.48) for high school education, 1.71 (lower bound: 1.67) for middle school education, and 1.78 (lower bound: 1.73) for elementary school education in overall sample (supplementary table S5). Therefore, unmeasured confounders with a considerable magnitude would be needed to completely nullify the observed association. The sensitivity analyses using multiple imputation confirmed the similar finding that as the level of education decreases, the OR of the indirect effect increased (supplementary table S6). The OR of the mediating effect and proportion mediated were greater in the 2020 sample than 2017 sample (supplementary table S7).

## Discussion

This study has shown how educational differences can contribute to disparities in subjective well-being within the theoretical framework of precarious employment. Additionally, we believe our study makes a meaningful contribution to the literature by exploring, for the first time, the mediating role of MEP in the relationship between educational attainment and psychological well-being of workers, especially in the Korean context. We observed that workers with lower levels of educational attainment were associated with an increase in MEP, which in turn was associated with the poor subjective well-being. Among Korean workers, MEP accounts for approximately 48.5–63.9% of the elevated OR of the poor subjective well-being observed in those with lower levels of education compared to those who have completed college education. Our findings suggest that insufficient educational attainment may result in workers having high level of MEP, thereby increasing the OR of having poor subjective well-being. Therefore, our study highlights the importance of MEP as a social determinant of poor subjective well-being and a significant contributor to mental health inequalities resulting from educational differences.

According to the literature, poor educational attainment can result in mental health deterioration through various pathways. For instance, individuals with lower educational attainment are more likely to experience several risk factors, such as lack of psychosocial resources (46), low self-efficacy (47), or lack of mental health literacy (48), which can be harmful to their well-being and mental health. Along with these factors, our findings revealed that MEP accounted for a significant portion of the effect of educational gradients on poor subjective well-being. This indicates that MEP serves as a key mediator between educational level and poor subjective well-being. Our findings are in line with previous studies that have explored the mediating role of work environments and in the education-health relationship (27, 28). A recent study conducted in the US has demonstrated that the influence of educational achievement on mental health problems is partially mediated through multidimensional employment quality, which accounts for approximately 32% of the total effect (35). The estimated mediating role of MEP was approximately 48–64% in this study, implying that precarious employment may have a larger contribution to the disparity in well-being associated with educational attainment within the Korean context compared with other countries. Additionally, as the level of education decreases, the indirect effect through MEP increases in a dose-dependent manner, while the proportion mediated was relatively lower among workers with middle school or elementary school education. This may be attributable to the fact that individuals with lower levels of education are more likely to concurrently experience other risk factors (eg, social resources, mental health literacy), in addition to employment precariousness.

Based on theoretical pathways explaining how MEP affects workers’ subjective well-being, various experiences of MEP, such as low wages, employment insecurity, and temporal uncertainty, can lead to negative economic, relational, and behavioral responses. These negative responses include material hardship, presentism, and work-family conflict, which can ultimately result in the deterioration of mental health (49). Moreover, a recent mediation analysis conducted by Rivero et al (17) suggested that European workers with precarious jobs were more likely to be exposed to psychosocial risk factors, such as lack of social support and high job demands with little control, which contributed to the deterioration of having poor subjective well-being. Our findings are also consistent with those of previous studies that have demonstrated that MEP is positively associated with poor mental health, including chronic stress, depression, and psychotropic drug use (18, 22, 50–52).

When examining the gendered results of the association between educational attainment and MEP, we found that MEP was higher among female workers. Several recent studies conducted in different regional contexts, such as in Europe and the USA, have also indicated higher levels of MEP among women (53, 54). Interestingly, while women have higher levels of MEP than men, the indirect effect of education on subjective well-being was found to be stronger for men. Previous studies have suggested that highly educated women may opt out of decent job positions because of gender-biased family responsibilities (55, 56). In Korea, women are often burdened with disproportionate housework and caregiving responsibilities, which can force them to take time off from their careers and work part-time, ultimately contributing to an increase in MEP (55). In addition, Cho et al (57) have argued in their recent study that hiring discrimination in the labor market can prevent women with high educational attainment from accessing decent and well-paying work opportunities. Therefore, due to the overall increase in MEP among highly educated women, the indirect effect of education on subjective well-being that is mediated through MEP may be less pronounced for women than for men.

Our study has some limitations. First, although we have utilized the causal mediation nomenclature of “effect” in order to enhance clarity, the true causal relationship between educational attainment, precarious employment, and subjective well-being could not be fully asserted due to the observational nature of this study. We could not rule out the possibility of the effect of unmeasured confounders, such as prior psychiatric disorders or parental socio-economic status (supplementary figure S2), as well as the possibility of reverse causation, in which poor subjective well-being may affect employees’ probability to be employed in highly precarious jobs. Second, workers with lower levels of education are vulnerable to layoffs and face limited opportunities for labor market entry. As this study is based on the cross-sectional design and primarily focuses on the employment precariousness among workers, those who were unemployed were excluded from our analysis, which may lead to the selection bias (Figure S2). Therefore, our findings cannot be generalized to the extent to which educational attainment contributes to the gradient of subjective well-being in the whole population. Further longitudinal studies should be followed to fully understand how education disparities can contribute to mental health gradients through unemployment. Third, our data contains a substantial proportion of missing values. To address this issue, we employed a multiple imputation as a sensitivity analysis, which shows the similar results as our main analysis. However, despite these approaches, there is a possibility of biased estimation as a result of violation of MAR assumption. Fourth, the relationship between MEP and mental health varies depending on regional contexts, including variations in occupational safety and health policies, as well as cultural differences (58). Therefore, the observed findings may not necessarily be generalizable or applicable to other regions or countries. Fifth, the association between educational attainment and MEP can vary depending on social and economic conditions, as well as labor policies. For example, our sensitivity analysis reveals that the indirect effect of low educational attainment on subjective well-being, mediated through MEP, intensified during the COVID-19 pandemic. This implies that COVID-19 may have disproportionately elevated the precariousness experienced by workers with lower levels of education. Sixth, our research outcome should be interpreted in terms of the workers’ psychological well-being, and therefore does not necessarily imply clinical mental health problems such as depression, anxiety disorders, or suicidality. Future research is needed to investigate how MEP mediates the relationship between educational attainment and psychiatric disorders.

### Concluding remarks

Our study provides evidence that the relationship between workers’ educational attainment and their mental health is partially mediated by MEP, indicating that MEP may account for a substantial proportion of the educational gradients in subjective well-being among workers. By highlighting the inequality in precarious employment according to educational level, as well as the importance of providing decent work to improve the mental health of the working population, we believe that our findings contribute to the literature and inform policy. Policies aimed at reducing MEP at both the structural and organizational levels are needed to improve workers’ well-being.

## Supplementary material

Supplementary material
