# Supplementary material for: Multidimensional employment precariousness mediates the association between low educational attainment and poor subjective well-being: results from a nationwide cross-sectional study in South Korea
Source: Scand J Work Environ Health. 2023 Sep 29;49(7):506–17. doi: 10.5271/sjweh.4109 (PMC10833203; doi:10.5271/sjweh.4109)
Supplement: Supplementary material [file SJWEH-49-506-S001.pdf]

Multidimensional employment precariousness mediates the association between low educational attainment and poor subjective well-being: results from a nationwide cross-sectional study in South Korea[1]

by Seong-Uk Baek, MD, Min-Seok Kim, MD, Myeong-Hun Lim, MD, Taeyeon Kim, MD, Jong-Uk Won, PhD, Jin-Ha Yoon, PhD [2]

1. *Supplementary Materials*
2. *Correspondence to: Jin-Ha Yoon, MD, PhD, Department of Preventive Medicine, Yonsei University College of Medicine, Yonsei University Health System, 50-1 Yonsei-ro, Seodaemun-gu, Seoul 03722, Republic of Korea. [E-mail: flyinyou@yuhs.ac]*

Figure S1. Distribution of multidimensional employment precariousness according to gender

Figure S2. Directed acyclic graph of the association between educational attainment, precarious employment, and subjective well-being

Table S1. Multidimensional employment precariousness according to study variables

Table S2. Prevalence of poor subjective well-being according to study variables

Table S3. Distribution of characteristics according to outcome status

Table S4. Effect modification by gender in two indirect paths

Table S5. Sensitivity analysis. E-values were calculated on the risk ratio scale

Table S6. Mediating effect of multidimensional employment precariousness on the association between low educational attainment and poor subjective well-being. Multiple imputation was used to handle missing values

Table S7. Mediating effect of multidimensional employment precariousness on the association between low educational attainment and poor subjective well-being separated by survey year

Supplementary details. Counterfactual-based mediational analysis employed in this study

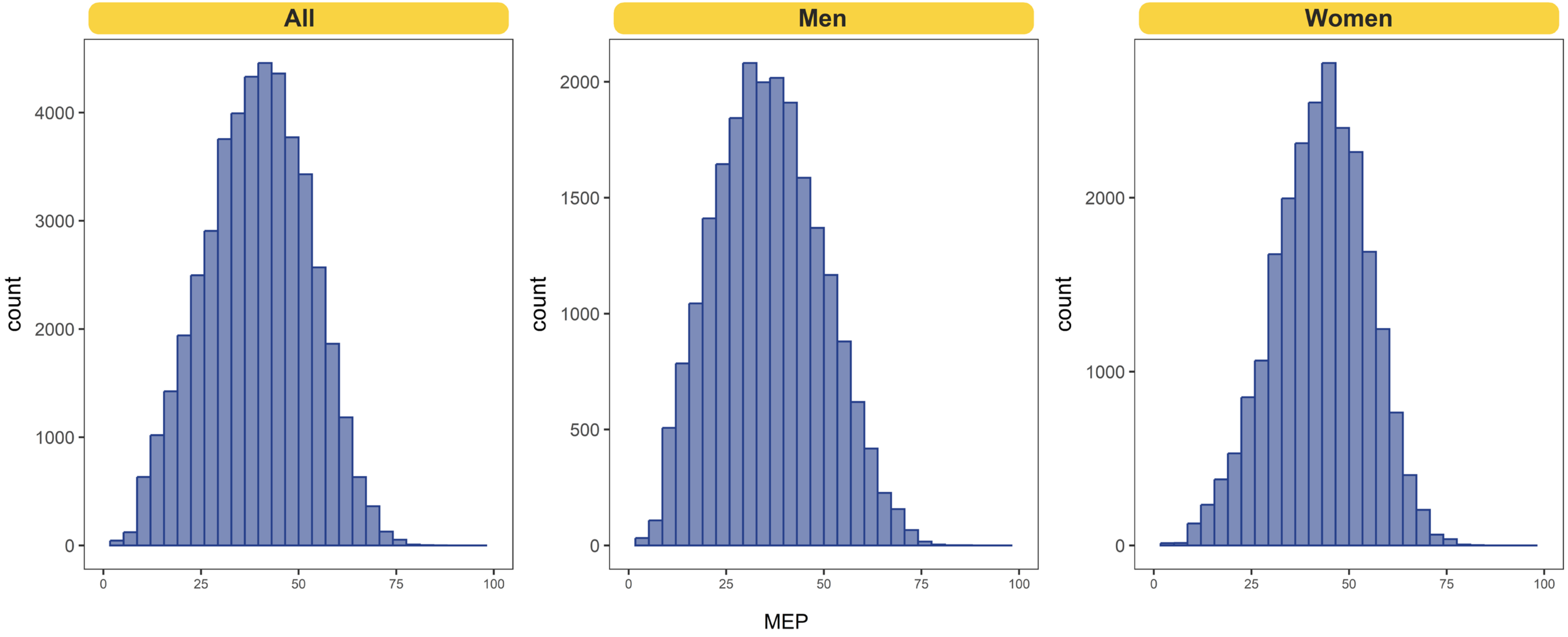

Figure S1. Distribution of multidimensional employment precariousness according to gender (MEP: Multidimensional Employment Precariousness)

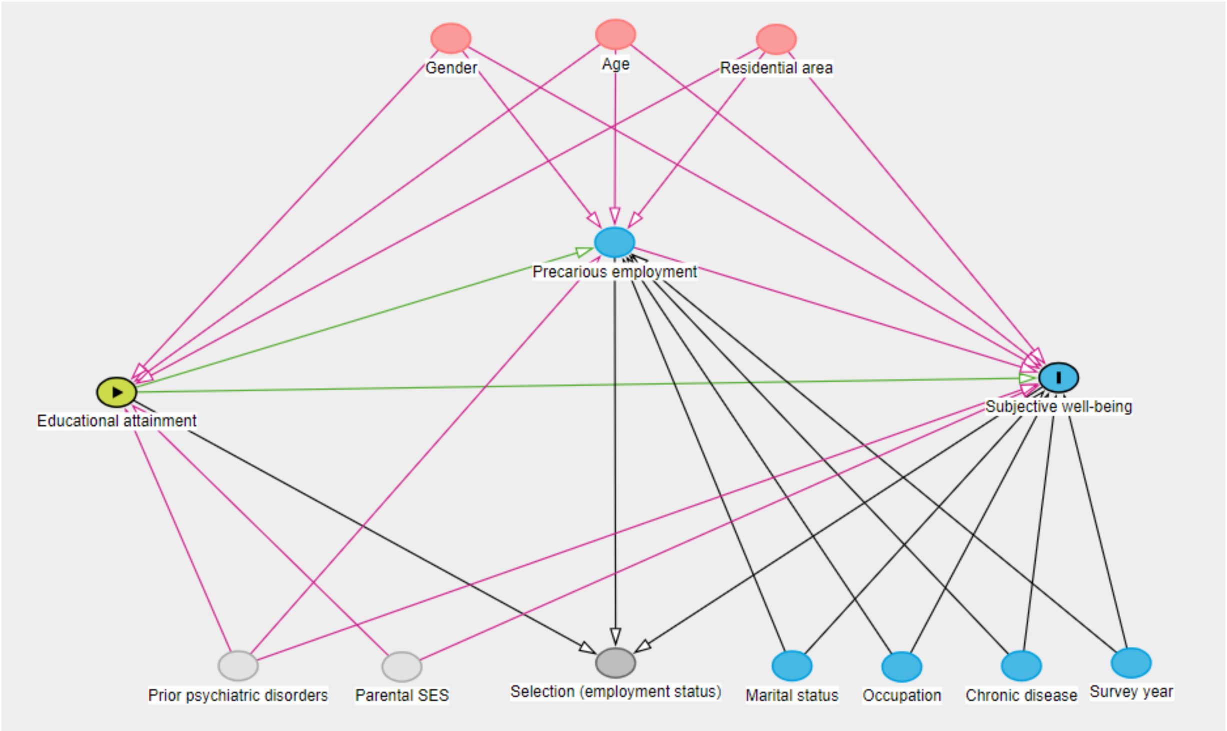

Figure S2. Directed acyclic graph of the association between educational attainment, precarious employment, and subjective well-being. The variables indicated by translucent gray circles represent potential unobserved confounders (prior psychiatric disorders, parental socioeconomic status), while the dark gray circles represent a selection process (employment status).

Table S1. Multidimensional employment precariousness according to study variables (SD: Standard Deviation).

Characteristics	All	Men	Women
	Mean (SD)	Mean (SD)	Mean (SD)
Mean (SD)	39.9 (12.7)	36.4 (13.0)	43.1 (11.4)
Educational attainment			
College or above	36.0 (12.1)	32.7 (12.0)	39.5 (11.2)
High school	44.3 (11.5)	41.5 (12.4)	46.6 (10.2)
Middle school	49.5 (10.1)	48.0 (11.3)	50.5 (9.2)
Elementary school or below	51.1 (10.0)	49.9 (11.3)	51.7 (9.1)
Age group			
19–29	46.2 (10.9)	45.8 (11.2)	46.5 (10.5)
30–39	37.7 (11.1)	35.5 (10.9)	40.2 (10.7)
40–49	37.0 (12.7)	32.1 (12.0)	41.4 (11.6)
50–59	39.6 (13.3)	33.9 (13.5)	43.7 (11.5)
60–65	45.0 (12.3)	42.5 (13.7)	47.4 (10.4)
Residential area			
Metropolitan	41.2 (13.9)	36.8 (13.0)	42.9 (11.4)
Small cites/rural	39.8 (12.6)	36.0 (13.0)	43.3 (11.3)
Marital status			
Married	38.5 (12.9)	33.8 (12.8)	42.6 (11.5)
Unmarried or others	42.6 (11.7)	41.1 (12.1)	44.1 (11.1)
Occupation			
Blue collar	42.9 (12.4)	40.4 (12.5)	48.4 (10.2)
Service and sales worker	45.7 (11.1)	42.2 (12.7)	47.1 (10.1)
White collar	34.6 (11.5)	30.4 (11.1)	38.1 (10.7)
Chronic disease			
Yes	41.2 (13.9)	37.5 (14.6)	44.4 (12.4)
No	39.8 (12.6)	36.4 (13.0)	43.0 (11.3)
Survey year			
2017	42.4 (13.0)	38.5 (13.3)	45.9 (11.5)
2020	37.0 (11.7)	34.0 (12.3)	39.8 (10.3)

Table S2. Prevalence of poor subjective well-being according to study variables. Values are presented as N (row percentages, %).

	All		Men		Women	
	Poor subjective well-being		Poor subjective well-being		Poor subjective well-being	
	No	Yes	No	Yes	No	Yes
Educational attainment						
College or above	20,630 (76.0)	6499 (24.0)	10599 (76.3)	3290 (23.7)	10031 (75.8)	3209 (24.2)
High school	11,545 (68.7)	5267 (31.3)	4953 (66.7)	2476 (33.3)	6592 (70.3)	2791 (29.7)
Middle school	1333 (59.4)	910 (40.6)	498 (56.7)	380 (43.3)	835 (61.2)	530 (38.8)
Elementary school or below	406 (55.2)	329 (44.8)	141 (54.9)	116 (45.1)	265 (55.4)	213 (44.6)
Age group						
19–29	5461 (78.0)	1544 (22.0)	2686 (78.4)	740 (21.6)	2775 (77.5)	804 (22.5)
30–39	8553 (75.0)	2854 (25.0)	4528 (74.7)	1536 (25.3)	4025 (75.3)	1318 (24.7)
40–49	9186 (72.5)	3481 (27.5)	4334 (72.0)	1687 (28.0)	4852 (73.0)	1794 (27.0)
50–59	8149 (69.3)	3618 (30.7)	3420 (68.4)	1580 (31.6)	4729 (69.9)	2038 (30.1)
60–65	2565 (63.0)	1508 (37.0)	1223 (63.0)	719 (37.0)	1342 (63.0)	789 (37.0)
Residential area						
Metropolitan	17,610 (74.5)	6027 (25.5)	8332 (74.7)	2822 (25.3)	9278 (74.3)	3205 (25.7)
Small cities/rural	16,304 (70.0)	6978 (30.0)	7859 (69.6)	3440 (30.4)	8445 (70.5)	3538 (29.5)
Marital status						
Married	22,532 (72.7)	8481 (27.3)	10,552 (72.4)	4017 (27.6)	11980 (72.9)	4464 (27.1)
Unmarried or others	11,382 (71.6)	4524 (28.4)	5639 (71.5)	2245 (28.5)	5743 (71.6)	2279 (28.4)
Occupation						
Blue collar	8948 (66.0)	4617 (34.0)	6197 (66.2)	3162 (33.8)	2751 (65.4)	1455 (34.6)
Service and sales worker	8949 (72.7)	3358 (27.3)	2682 (76.1)	844 (23.9)	6267 (71.4)	2514 (28.6)
White collar	16,017 (76.1)	5030 (23.9)	7312 (76.4)	2256 (23.6)	8705 (75.8)	2774 (24.2)
Chronic disease						
Yes	1198 (51.4)	1132 (48.6)	553 (50.7)	537 (49.3)	645 (52.0)	595 (48.0)
No	32,716 (73.4)	11,873 (26.6)	15638 (73.2)	5725 (26.8)	17,078 (73.5)	6148 (26.5)

Survey year						
2017	18,497 (73.8)	6583 (26.2)	8880 (73.8)	3146 (26.2)	9617 (73.7)	3437 (26.3)
2020	15,417 (70.6)	6422 (29.4)	7311 (70.1)	3116 (29.9)	8106 (71.0)	3306 (29.0)

\

Table S3. Distribution of characteristics according to outcome status. Values are presented as N (%). (MEP: Multidimensional Employment Precariousness).

Characteristics	All		Men		Women	
	Poor subjective well-being		Poor subjective well-being		Poor subjective well-being	
	No	Yes	No	Yes	No	Yes
MEP						
Mean (SD)	38.8 (12.6)	42.6 (12.5)	35.2 (12.8)	39.5 (13.2)	42.1 (11.4)	45.5 (11.1)
Educational attainment						
College or above	20630 (60.8)	6499 (50.0)	10599 (65.5)	3290 (52.5)	10031 (56.6)	3209 (47.6)
High school	11545 (34.0)	5267 (40.5)	4953 (30.6)	2476 (39.5)	6592 (37.2)	2791 (41.4)
Middle school	1333 (3.9)	910 (7.0)	498 (3.1)	380 (6.1)	835 (4.7)	530 (7.9)
Elementary school or below	406 (1.2)	329 (2.5)	141 (0.9)	116 (1.9)	265 (1.5)	213 (3.2)
Age group						
19–29	5461 (16.1)	1544 (11.9)	2686 (16.6)	740 (11.8)	2775 (15.7)	804 (11.9)
30–39	8553 (25.2)	2854 (21.9)	4528 (28.0)	1536 (24.5)	4025 (22.7)	1318 (19.5)
40–49	9186 (27.1)	3481 (26.8)	4334 (26.8)	1687 (26.9)	4852 (27.4)	1794 (26.6)
50–59	8149 (24.0)	3618 (27.8)	3420 (21.1)	1580 (25.2)	4729 (26.7)	2038 (30.2)
60–65	2565 (7.6)	1508 (11.6)	1223 (7.6)	719 (11.5)	1342 (7.6)	789 (11.7)
Residential area						
Metropolitan	17610 (51.9)	6027 (46.3)	8332 (51.5)	2822 (45.1)	9278 (52.4)	3205 (47.5)
Small cites/rural	16304 (48.1)	6978 (53.7)	7859 (48.5)	3440 (54.9)	8445 (47.6)	3538 (52.5)
Marital status						
Married	22532 (66.4)	8481 (65.2)	10552 (65.2)	4017 (64.1)	11980 (67.6)	4464 (66.2)
Unmarried or others	11382 (33.6)	4524 (34.8)	5639 (34.8)	2245 (35.9)	5743 (32.4)	2279 (33.8)
Occupation						
Blue collar	8948 (26.4)	4617 (35.5)	6197 (38.3)	3162 (50.5)	2751 (15.5)	1455 (21.6)
Service and sales worker	8949 (26.4)	3358 (25.8)	2682 (16.6)	844 (13.5)	6267 (35.4)	2514 (37.3)
White collar	16017 (47.2)	5030 (38.7)	7312 (45.2)	2256 (36.0)	8705 (49.1)	2774 (41.1)
Chronic disease						
Yes	1198 (3.5)	1132 (8.7)	553 (3.4)	537 (8.6)	645 (3.6)	595 (8.8)
No	32716 (96.5)	11873 (91.3)	15638 (96.6)	5725 (91.4)	17078 (96.4)	6148 (91.2)
Survey year						
2017	18497 (54.5)	6583 (50.6)	8880 (54.8)	3146 (50.2)	9617 (54.3)	3437 (51.0)
2020	15417 (45.5)	6422 (49.4)	7311 (45.2)	3116 (49.8)	8106 (45.7)	3306 (49.0)

Table S4. Effect modification by gender in two indirect paths (MEP: Multidimensional Employment Precariousness; OR: Odds Ratio; CI: Confidence Interval)

**Path**	**All**	
	β	**95% CI**
**Model 1**		
Educational attainment → MEP		
College or above	0.00	Reference
High school	5.41	5.09–5.73
Middle school	11.92	11.19–12.66
Elementary school or below	13.77	12.49–15.05
Men	0.00	Reference
Women	7.30	7.05–7.55
Women × College or above	0.00	Reference
Women × High school	-0.99	-1.39 – -0.59
Women × Middle school	-4.85	-5.75 – -3.96
Women × Elementary school or below	-5.83	-7.39 – -4.28
	**All**	
	**OR**	**95% CI**
**Model 2**		
MEP → Poor mental health		
MEP (range 0–100)	1.03	1.03–1.03
Men	1.00	Reference
Women	0.82	0.70–0.95
Women × MEP	1.00	1.00–1.00

Models adjusted for gender, age, residential area, marital status, occupation, chronic disease, and survey year

Table S5. Sensitivity analysis. E-values were calculated on the risk ratio scale.

	Direct effect		Indirect effect	
	E-value for point estimate	E value for lower bound	E-value for point estimate	E value for lower bound
**All**				
High school	1.34	1.24	1.51	1.48
Middle school	1.75	1.60	1.71	1.67
Elementary school or below	1.82	1.57	1.78	1.73
**Men**				
High school	1.44	1.32	1.55	1.52
Middle school	1.79	1.56	1.83	1.77
Elementary school or below	1.70	1.31	1.92	1.83
**Women**				
High school	1.22	1.00	1.44	1.42
Middle school	1.69	1.49	1.61	1.56
Elementary school or below	1.84	1.54	1.66	1.60

The E-values for the point estimate and lower bound were derived from the odds ratios and their lower bound values presented in Table 3 of the main document, respectively.

Table S6. Mediating effect of multidimensional employment precariousness on the association between low educational attainment and poor subjective well-being. Multiple imputation was used to handle missing values. (N=57,569; OR: Odds Ratio; CI: Confidence Interval).

Educational attainment	Total effect	Direct effect	Indirect effect	Proportion mediated
	OR (95% CI)	OR (95% CI)	OR (95% CI)	% (95% CI)
**All**				
College or above	Reference	Reference	Reference	Reference
High school	1.46 (1.39–1.53)	1.17 (1.12–1.23)	1.24 (1.22–1.26)	57.6 (50.0–65.1)
Middle school	2.29 (2.09–2.51)	1.61 (1.48–1.77)	1.42 (1.38–1.46)	42.3 (37.0–47.6)
Elementary school or below	2.39 (2.07–2.76)	1.62 (1.40–1.87)	1.47 (1.42–1.53)	44.6 (36.6–52.6)
**Men**				
College or above	Reference	Reference	Reference	Reference
High school	1.63 (1.53–1.74)	1.29 (1.21−1.38)	1.27 (1.24–1.30)	48.3 (40.8–55.8)
Middle school	2.71 (2.37–3.11)	1.80 (1.57–2.06)	1.51 (1.44−1.57)	41.0 (34.5–47.5)
Elementary school or below	2.48 (1.97–3.12)	1.57 (1.25−1.97)	1.58 (1.49–1.68)	50.4 (36.1–64.8)
**Women**				
College or above	Reference	Reference	Reference	Reference
High school	1.33 (1.24–1.41)	1.09 (1.03–1.17)	1.21 (1.19–1.24)	68.2 (51.6–84.8)
Middle school	2.00 (1.77–2.25)	1.48 (1.31–1.67)	1.35 (1.31–1.40)	43.5 (35.1–51.9)
Elementary school or below	2.23 (1.86–2.69)	1.60 (1.33–1.92)	1.40 (1.34–1.46)	41.8 (31.2–52.5)

Models adjusted for gender, age, residential area, marital status, occupation, chronic disease, and survey year.

Table S7. Mediating effect of multidimensional employment precariousness on the association between low educational attainment and poor subjective well-being, separated analysis by survey year. (N=46,919; OR: Odds Ratio; CI: Confidence Interval).

Educational attainment	Total effect		Direct effect		Indirect effect		Proportion mediated	
	2017	2020	2017	2020	2017	2020	2017	2020
	OR (95% CI)	OR (95% CI)	OR (95% CI)	OR (95% CI)	OR (95% CI)	OR (95% CI)	% (95% CI)	% (95% CI)
**All**								
College or above	Reference	Reference	Reference	Reference	Reference	Reference	Reference	Reference
High school	1.42 (1.33–1.53)	1.46 (1.35–1.58)	1.16 (1.08–1.24)	1.12 (1.04–1.21)	1.23 (1.21–1.26)	1.30 (1.27–1.34)	59.2 (45.9–72.4)	69.8 (55.2–84.5)
Middle school	2.20 (1.93–2.51)	2.07 (1.77–2.43)	1.60 (1.40–1.82)	1.35 (1.15–1.58)	1.38 (1.33–1.43)	1.54 (1.48–1.60)	40.5 (32.8–48.3)	59.3 (44.9–73.8)
Elementary school or below	2.24 (1.81–2.77)	2.54 (1.97–3.28)	1.58 (1.29–1.95)	1.52 (1.19–1.95)	1.41 (1.36–1.47)	1.67 (1.58–1.77)	42.9 (30.4–55.4)	55.0 (39.1–71.0)
**Men**								
College or above	Reference	Reference	Reference	Reference	Reference	Reference	Reference	Reference
High school	1.55 (1.40–1.72)	1.66 (1.50–1.85)	1.26 (1.13–1.40)	1.17 (1.05–1.29)	1.24 (1.19–1.28)	1.43 (1.37–1.48)	41.1 (29.3–62.0)	69.7 (54.4–85.0)
Middle school	2.36 (1.92–2.89)	2.44 (1.90–3.13)	1.66 (1.35–2.03)	1.35 (1.05–1.73)	1.42 (1.34–1.51)	1.81 (1.68–1.95)	41.1 (29.3–52.9)	66.6 (45.6–87.6)
Elementary school or below	2.24 (1.57–3.20)	2.66 (1.73–4.07)	1.54 (1.09–2.17)	1.29 (0.85–1.96)	1.46 (1.36–1.57)	2.05 (1.84–2.30)	46.8 (19.1–74.6)	73.7 (30.6–116.8)
**Women**								
College or above	Reference	Reference	Reference	Reference	Reference	Reference	Reference	Reference
High school	1.30 (1.17–1.45)	1.31 (1.18–1.47)	1.07 (0.97–1.19)	1.09 (0.97–1.20)	1.21 (1.18–1.25)	1.22 (1.18–1.25)	73.8 (40.3–100.7)	72.2 (37.1–100.7)
Middle school	2.06 (1.71–2.48)	1.76 (1.43–2.17)	1.55 (1.29–1.38)	1.29 (1.05–1.58)	1.32 (1.27–1.38)	1.37 (1.31–1.44)	38.8 (27.3–50.4)	55.4 (31.9–78.9)
Elementary school or below	2.21 (1.67–2.92)	2.29 (1.65–3.17)	1.63 (1.23–2.15)	1.58 (1.14–2.17)	1.36 (1.29–1.43)	1.45 (1.36–1.55)	38.5 (21.3–55.7)	45.1 (20.3–69.9)

Models adjusted for gender, age, residential area, marital status, occupation, and chronic disease

Supplementary details. Counterfactual-based mediational analysis employed in this study

The causal mediation analysis decomposed the total effect into direct effect (educational attainment [x] → poor subjective well-being [y]) and indirect effect (educational attainment [x] → MEP score [z] → poor subjective well-being [y]). The decomposition of the total effect can be estimated by the following equation[1]:

$$\text{In}\left(\frac{O_{x=1,z|x=1}}{O_{x=0,z|x=0}}\right)\text{[Total effect]} = \text{In}\left(\frac{O_{x=0,z|x=1}}{O_{x=0,z|x=0}}\right)\text{[Indirect effect]} + \text{In}\left(\frac{O_{x=1,z|x=1}}{O_{x=0,z|x=1}}\right)\text{[Direct effect]}$$

In the above equation, the odds of having a poor subjective well-being is presented as O. The left-hand side of the equation (total effect) denotes the odds when the education level, denoted as x, takes on a specific value (x=1), divided by the odds when x represents the reference education level (college or above) (x=0). The division is conditioned on the distribution of the mediator (MEP score) in each education group ($z|x = 1$ or $z|x = 0$).

The first term on the right side of the equation corresponds to the odds under a counterfactual situation where x is the reference group but assumes the mediator distribution of the specific education group ($O_{x=0,z|x=1}$). This term is divided by the odds when x represents the reference group, given the distribution of the mediator of the reference education level ($O_{x=0,z|x=0}$). Therefore, it quantifies the indirect effect, which can be interpreted as the effect of educational attainment on poor subjective well-being transmitted through high MEP score.

The second term on the right side of the equation represents the odds when x represents the specific education group, given the distribution of the mediator of the specific education level ($O_{x=1,z|x=1}$). This term is divided by the odds in a counterfactual situation where x is the reference group but assumes the mediator distribution of the specific education group ($O_{x=0,z|x=1}$). Therefore,

[1] Buis ML. Direct and indirect effects in a logit model. Stata J. 2010 Winter;10(1):11-29.

it quantifies the direct effect, which can be interpreted as the effect of educational attainment on poor subjective well-being, transmitted through all other potential mechanisms.

For these estimates of direct and indirect effects to be interpreted causally, following assumptions should be met:

(i) No unmeasured confounding of the exposure-outcome relationship; (ii) No unmeasured confounding of the mediator-outcome effect; (iii) No unmeasured confounding of the exposure-mediator effect; (iv) No downstream effect of the exposure that confounds mediator-outcome effect.
